# Mutational signatures and the genomic landscape of betel quid chewing‐associated tongue carcinoma

**DOI:** 10.1002/cam4.1888

**Published:** 2019-01-22

**Authors:** Weilong Zhang, Mu Wang, Qifeng Wu, Qing Zhu, Yuchen Jiao, Yiming Zhu, Beibei Yang, Song Ni, Jianjun Yu, Hong Sun, Yi‐Xin Zeng

**Affiliations:** ^1^ National Cancer Center/National Clinical Research Center for Cancer/Cancer Hospital Chinese Academy of Medical Sciences and Peking Union Medical College Beijing China; ^2^ State Key Lab of Molecular Oncology, National Cancer Center/Cancer Hospital Chinese Academy of Medical Sciences and Peking Union Medical College Beijing China; ^3^ Department of Otolaryngology Head and Neck Surgery, Xiangya Hospital Central South University Changsha Hunan China; ^4^ Department of Stomatology, Peking Union Medical College Hospital Chinese Academy of Medical Sciences & Peking Union Medical College Beijing China; ^5^ Laboratory of Cell and Molecular Biology, National Cancer Center/Cancer Hospital Chinese Academy of Medical Sciences and Peking Union Medical College Beijing China; ^6^ Department of Head and Neck Surgery, National Cancer Center/Cancer Institute and Hospital Chinese Academy of Medical Sciences and Peking Union Medical College Beijing China; ^7^ Department of Head and Neck Surgery, Hunan Cancer Hospital and The Affiliated Cancer Hospital of Xiangya School of Medicine Central South University Changsha Hunan China; ^8^ Department of Experimental Research, Sun Yat‐sen University Cancer Center, State Key Laboratory of Oncology in Southern China, Collaborative Innovation Center for Cancer Medicine Guangzhou Guangdong Province China

## Abstract

Our study presents the genetic landscape betel quid chewing‐associated tongue carcinomas (BQ‐TCs). We compared the genetic landscape and mutational signatures of 15 BQ‐TCs, five nonbetel quid chewing‐associated tongue carcinomas (nBQ‐TCs), and 82 tongue carcinomas in general population from the TCGA (TCGA‐TCs) project. The highlights of this research mainly include: (a) The genetic landscape of BQ‐TC was characterized with frequent mutations in RASA1 gene and in CpG islands throughout the genome. (b) The BQ‐TC had a distinct mutational signature from that of nBQ‐TC and tongue carcinomas in the general population, and this signature was associated with the mutations in RASA1 and in CpG islands. (c) Our study indicates that betel quid (BQ) chewing classifies a distinct group of tongue carcinoma. The BQ chewing might not contribute to the tumorigenesis of tongue carcinomas as a mutagen.

## 1 INTRODUCTION

Areca is the fourth legal additive compound in the world, just next to alcohol, smoking, and caffeine. There are 600‐1200 million users of areca products globally.^1^, ^2^, ^3^ Consumption of fresh betel nut (BN) or betel quid (BQ), which is betel product containing a large variety of ingredients, is common in South and Southeast Asia as well as in migrant Asian communities around the world. Fifty‐eight percent of the 390 thousand new oral cancer (OC) cases in the world are enriched in South and Southeast Asia with areca chewing (BN chewing and BQ chewing) habits, and this risk factor could be the dominant cause of OCs in these populations.^4^

In 2004, the International Agency of Research on Cancer (IARC) reported that habitual BN chewing is associated with oral precancerous lesion and OC, while BQ chewing could cause OCs, pharynx cancer, and esophageal cancer.^3^, ^5^ In previous studies, alkaloid‐related DNA adducts, DNA strand breaks, and reactive oxygen species could be possible mechanisms of areca‐associated OC.^6^, ^7^, ^8^, ^9^ Carcinogen‐related DNA adducts or DNA damage might play an important role during BQ chewing‐associated tumorigenesis from experiment model.^10^

However, the BQ product could contain a complex composition, with various ingredients added during the production process from fresh nut to BQ product. There are debates that the tumorigenesis should not be attributed to the areca nut itself; instead, the ingredients improperly added in the production process play the major tumorigenic role.^5^ There are also arguments that the tobacco added in BQ product is the dominant tumorigenic factor.^6^ In addition, HPV infection has also been shown to be an important factor in the development of tongue cancer.^6^, ^11^

In this study, we performed genomic study on betel quid chewing‐associated tongue cancer (BQ‐TC) from Hunan Province. BQ chewing is common in Hunan Province, and the prevalence of BQ chewing is as high as 64.5%‐82.7% in some regions.^12^, ^13^ The incidence of OC in the Hunan Province is significantly higher than that in other provinces in China. Different from Southeast Asian countries, tobacco is not added in the BQ. By whole‐genome and whole‐exome sequencing, we studied the genetic features of BQ‐TC and found that BQ chewing defined a distinct group of tongue cancer with characteristic mutational signature and frequent mutations in RASA1 gene and CpG islands.

## 2 MATERIALS AND METHODS

### 2.1 Subjects and tissue samples

Our study included 20 patients with lingual carcinoma who underwent diagnosis and treatment with surgical resection followed by postoperative adjuvant therapy, primary radiotherapy, or concurrent chemoradiotherapy between 2014 and 2015 at the Head and Neck Oncology Department, Hunan Cancer Hospital & The Affiliated Cancer Hospital of Xiangya School of Medicine, Central South University, Changsha, China (15 BQ‐TC), or Cancer Hospital Chinese Academy of Medical Science, Beijing, China (five nBQ‐TC). Specimens were collected prior to radiotherapy and chemotherapy. The patients underwent clinical staging of their cancer according to the 1997 American Joint Committee on Cancer system. Clinicopathological data including age, sex, smoking, BQ chewing, alcohol intake history, nodal status, tumor site, and outcome data were obtained retrospectively (Table S1 and Table S2). The study was approved by the Human Research Ethics Committee of Hunan Cancer Hospital & Ethics Committee of Cancer Hospital Chinese Academy of Medical Science. The sequencing data of 82 tongue carcinoma (TCGA‐TC) samples and 201 oral carcinoma (TCGA‐ORCA) samples were downloaded from The Cancer Genome Atlas (TCGA) level 3 data^14^ and are included in Table S3 and Table S4. The TCGA‐TC samples were assumed to be obtained from patients who lack BQ chewing habits, according to the clinical characteristics presented in Table S2. The BQ‐TC and nBQ‐TC samples we collected and all the TCGA tongue carcinomas we used are squamous carcinomas.

### 2.2 Whole‐exome sequencing and data analysis

Genomic DNA was extracted from 10 tumors and matched normal DNA samples with DNeasy Blood & Tissue Kit (cat# 69504; Qiagen, Hilden, Germany) following the manufacturer's protocol and sequenced the exome of approximately 21 000 protein‐coding genes (Table S5). We constructed genomic DNA libraries and capture the whole exome with the Agilent SureSelect v5 human exome kit and sequenced the captured libraries on the Illumina HiSeq genome analyzer with paired‐end 150 base reads as previously described.^15^

Somatic variants (SNV and indel) were identified from 10 pairs of BQ‐TC samples using GATK Best Practices pipeline.^16^, ^17^ Quality control of raw data was constructed with FastQC software (http://www.bioinformatics.babraham.ac.uk/projects/fastqc/). The sequencing reads were aligned to the reference of human genome hg19 using the BWA‐MEM algorithm from software.^18^ We marked PCR duplicates using Picard software (http://broadinstitute.github.io/picard/). Indel realignment of each bam files and base quality score recalibration were constructed with GATK (https://software.broadinstitute.org/gatk/). Somatic mutations were called using MuTect (version 1.1.4) following the criteria^19^: (a) It was supported by ≥3 distinct reads in tumor samples; (b) the proportion of distinct reads with a particular mismatched base was ≥5% of the total distinct reads in tumor samples; and (c) it was not present in>2 distinct reads in the matched normal sample. Indels were identified using VarScan (version 2.3.7)^20^ with parameter of somatic *P*‐value 0.05. Somatic variants (SNV and indel) of each sample were annotated with ANNOVAR (http://annovar.openbioinformatics.org/en/latest/) software.

### 2.3 Whole‐genome sequencing and data analysis

Ten pairs of genomic DNA from frozen matched tumor and normal samples were used to construct genomic library and were sequenced on an Illumina HiSeq X Ten sequencer with paired‐end 150 base reads. The average depth of each sample was 54X (range from 45 to 69) for the tumor and 36X (range from 26 to 49) for the normal samples. The percentage of mapped reads was 95.6% (range from 84.1% to 99.6%) (Table S6). Somatic mutations were called using MuTect (version 1.1.4)^19^ and the GATK Best Practices pipeline^16^, ^17^ (see whole‐exome sequencing and data analysis method). Indels were identified using VarScan (version 2.3.7)^20^ based on a somatic *P*‐value of 0.05. We used stringent filter criteria and applied a joint calling strategy to avoid false‐positive calls due to the relatively low sequencing depth in the normal samples of whole‐genome sequencing (WGS). In addition to the matched normal sample, the variants in each tumor sample were filtered against the pooled variants from all the normal samples. The quality of these mutations was manually reviewed with Integrated Genomics Viewer (IGV)^21^, ^22^ and was validated by Sanger sequencing on a subset of all somatic mutations.

### 2.4 Mutational signature analysis

The mutational signature of 15 BQ‐TC (whole‐exome sequencing or exome region), five nBQ‐TC (exome region), and 82 TCGA‐TC (whole‐exome sequencing) was analyzed using the Wellcome Trust Sanger Institute Mutational Signature Framework with the nonnegative matrix factorization (NMF) algorithm.^23^ Mutational signature analysis was conducted following this four‐step procedure:

- A 96 mutational‐class matrix, including the six mutation types (C>A, C>G, C>T, T>A, T>C, and T>G), 5′ context (C, A, G, T), and 3′ context (C, A, G, T), was built from mutation data of all samples.
- The number of processes operative in 15 BQ‐TC, five nBQ‐TC, and 82 TCGA‐TC samples was identified based on signature stability and Frobenius reconstruction errors obtained for *K* = 1 to 15 signatures.
- Mutational signatures (Sig A, B, and C) of all samples were deciphered using the NMF algorithm with the number of processes operative in step b.
- Unsupervised hierarchical clustering (R “stats” package, complete‐linkage algorithm) of the three mutational signatures (Sig A, B, and C) identified in our series with 30 mutational signatures (Sig 1‐30) previously identified in a pan‐cancer study^24^ was conducted using cosine similarity.

### 2.5 Copy number analysis of whole‐genome sequencing

Somatic copy number analyses of five BQ‐TCs and five nBQ‐TCs were conducted from WGS data of tumors and matched normal samples using Control‐FREEC software (https://omictools.com/control-freec-tool).^25^, ^26^ The copy number profiles were normalized using GC content. Recurrence of chromosomal alterations in BQ‐TC and nBQ‐TC was calculated with cghMCR (version 1.28.0) from WGS data.^27^ SGOL scores for each segmented data (*y*‐axis) plotted aligned along the *x*‐axis in genome order. Green represents chromosomal gain, and red denotes chromosomal loss.

### 2.6 HPV integration analysis

Human genome hg19 was downloaded from NCBI, and reads were mapped by BWA using the VirusFinder software (http://bioinfo.mc.vanderbilt.edu/VirusFinder/.).^28^ HPV integration analysis follows a four‐step procedure: (a) read subtraction (obtain unmapped reads), (b) virus detection from the unmapped reads, (c) if virus were detected in step b, virus integration site detection analysis was conducted, and (d) viral mutation detection. The confidence of HPV integrations was sorted by the supporting reads of pair break point reads and softclip reads.

### 2.7 Analysis of mutations in the CpG islands

The 28691 CpG island regions were obtained from UCSC (https://www.ucsc.edu/). The total size of the 28691 CpG island regions was approximately 22 Mb (about 0.7% of the whole genome). To test the enrichment for mutations on CpG island regions compared to the flanking regions, we compared the ratio of the total number of mutations to the total number of nucleotide positions in the CpG island regions (−50 to +50 nt) and in the flanking region (501‐1000 nt on either side, respectively) using a Fisher's exact test.^29^, ^30^ We performed this test and fold change for CpG island regions of six classes of mutation type (C>A, C>G, C>T, T>A, T>C, and T>G) and 16 classes of mutation type, respectively.

### 2.8 Gene expression in tongue carcinomas retrieved from the TCGA project

RNA‐seq data of 184 oral carcinoma tumor samples and 26 oral carcinoma tumor and matched normal samples were retrieved from the TCGA project. Gene expression was calculated using RPKM (reads per kilobase per million mapped reads). Data were analyzed with the Mann‐Whitney test and Wilcoxon matched‐pairs signed rank test and shown as the mean ± SEM. A *P*‐value of <0.05 was considered to be statistically significant. *RASA1* and APOBEC family gene expressions of each sample are summarized in Table S7‐9.

### 2.9 HPV detection in BQ‐TC and nBQ‐TC tumor samples through reverse dot blot

HPV detection in 15 BQ‐TC and five nBQ‐TC tumor samples was conducted through reverse dot blot method. HPV16, 18, 31, 33, 35, 39, 45, 51, 52, 53, 56, 58, 59, 66, 68, 73, 82 (high‐risk HPV) and HPV6, 11, 42, 43, 81, 83 (low‐risk HPV) were detected (totally 23 type of HPV) in this method. The whole detection follows a two‐step procedure: (a) the DNA of HPV was amplified with PCR of 40 cycles; and (b) DNA hybridization and HPV detection. The limit of HPV detection was 1.0 × 10^4^ copies/mL. A positive control was used in this detection.

R package "ggplot2" (https://cran.r-project.org/web/packages/ggplot2/) was used in this study. Data were expressed as the mean ± SEM in scatter plots for this paper. A statistical significance level of *P* < 0.05 was used.

## 3 RESULTS

### 3.1 Genome‐wide study on BQ‐TC and nBQ‐TC cases

**Figure 1:**
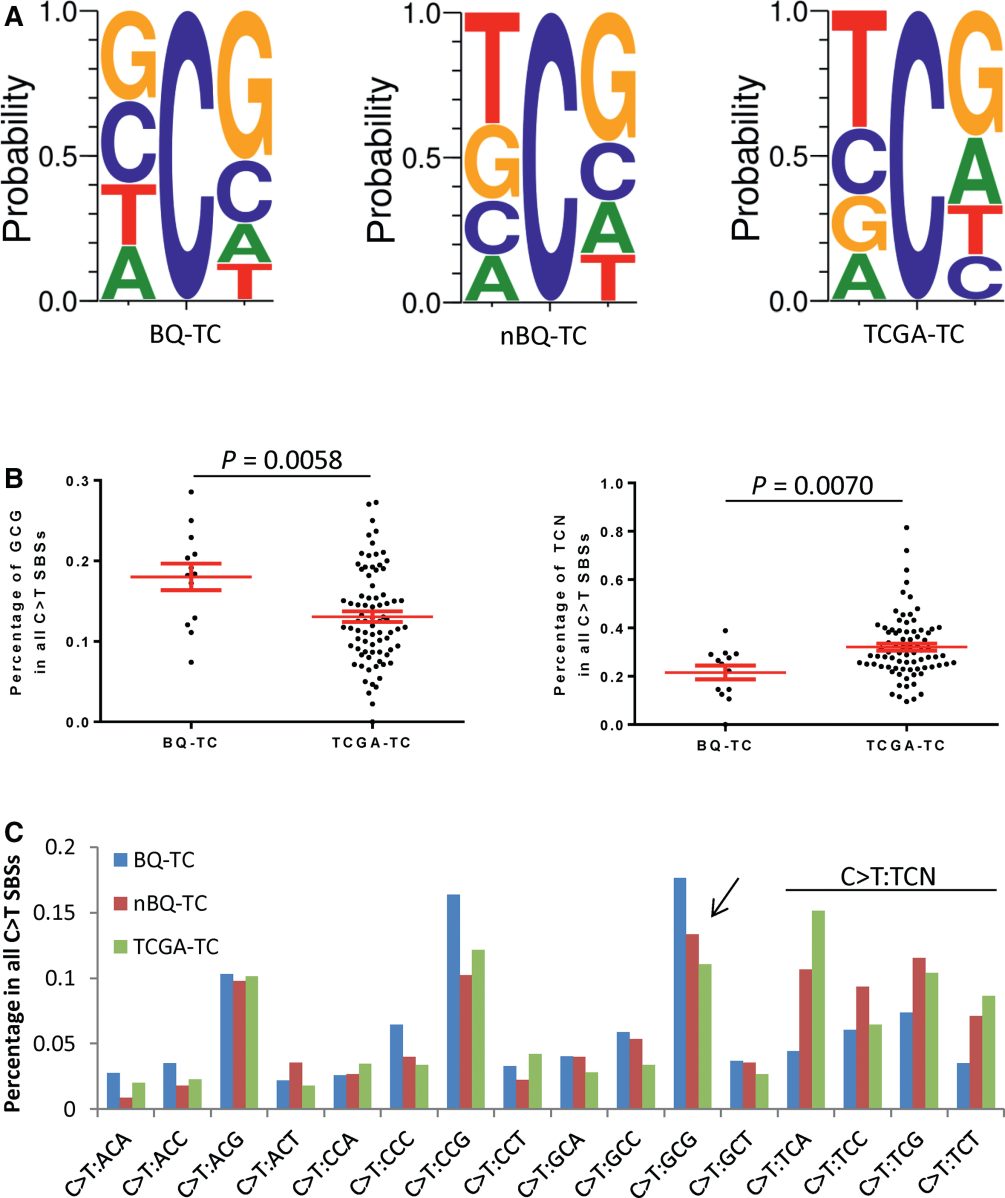
Mutational signature of the betel quid chewing‐associated tongue carcinoma (BQ‐TC) samples. A, The sequence contexts of the C>T mutations of the BQ‐TC, nonbetel quid chewing‐associated tongue carcinoma (nBQ‐TC), and tongue carcinomas from TCGA project (TCGA‐TC) samples. B, A scatter plot of the percentage of C>T mutations from the GCG (left) and TCN (right) (TCN: TCA, TCC, TCG, TCT) contexts in all of the C>T mutations in coding regions (unpaired *t* test, two‐sided). C, The context of the C>T mutations in the coding regions of the BQ‐TC, nBQ‐TC, and TCGA‐TC samples. For example, C>T:GCG indicates a C>T mutation in the context of GCG, where the C in the middle is changed to a T

We sequenced the genomes of 15 betel quid chewing‐associated tongue carcinoma (BQ‐TC) patients collected in the Hunan Province, and all the BQ‐TC patients included in this study were newly diagnosed and untreated patients with a clear history of BQ chewing (Table S1). Five nonbetel quid chewing‐associated tongue carcinoma (nBQ‐TC) tissue samples were obtained from newly diagnosed and untreated patients without a history of BQ chewing. We performed WGS on five BQ‐TC and five nBQ‐TC samples, and whole‐exome sequencing (WES) on additional 10 BQ‐TC samples (Figure 1A). We identified an average of 8056 somatic single‐base substitutions (SBSs) per genome or 70 per exome (Figure S1A). On the exome scale, the mutation load of BQ‐TC samples was similar to that of nBQ‐TC and TCGA‐TC (tongue carcinoma from TCGA project) samples (one‐way ANOVA) (Figure S1A). We compared the nonsynonymous mutation load of BQ‐TC with that in three tumor types that were associated with Group 1 carcinogens (Figure S1B).^31^, ^32^, ^33^ UV‐exposed melanomas (n = 7), smoking‐associated lung cancers (n = 10), and Helicobacter pylori‐associated gastric cancers (n = 8) harbored 336, 192, and 60 nonsynonymous SBSs per exome, respectively. The BQ‐TC patients (51 nonsynonymous SBSs per exome) showed a similar level to those with Helicobacter pylori‐associated gastric cancer (*P* > 0.05, one‐way ANOVA), and a much lower level than those with UV‐exposed melanoma (*P* < 0.0001, one‐way ANOVA) or smoking‐associated lung cancer (*P* = 0.0299, one‐way ANOVA) (Figure S1B).

### 3.2 The mutational signature of BQ‐TC

At exome level, the SBSs in the BQ‐TC samples exhibited a mutational signature with a dominant mutation pattern of C>T (equal to G>A) transitions (Figures S1C,D and S2). The C>T mutations showed no strand preference (Figure S1E). The BQ‐TC samples had a preference for a G in the position preceding the mutated C residue for both synonymous and nonsynonymous C>T mutations, while the nBQ‐TC and TCGA‐TC patients showed a strong preference for a T in that position (Figure 1A and S3A‐B). The presence of a G downstream of the mutated C residue was significantly more common in the C>T mutations of the BQ‐TC patients than in the nBQ‐TC and TCGA‐TC patients (Figure 1A‐C and S3A‐B). Thus, the BQ‐TC patients had a preference for GCG (to GTG) patterns in the C>T mutations (*P* = 0.0058, unpaired *t* test, two‐sided), while the TC (tongue carcinoma) cases reported in the TCGA database had a preference for a TCG (to TTG) pattern (*P* = 0.0070, unpaired *t* test, two‐sided) (Figure 1B‐C). The dominant differences between BQ‐TC and TCGA‐TC are GCG (to GTG) patterns (*P* = 0.0058, unpaired *t* test, two‐sided) and TCN (to TTN) patterns (*P* = 0.0070, unpaired *t* test, two‐sided) (Figure 1B‐C).

**Figure 2:**
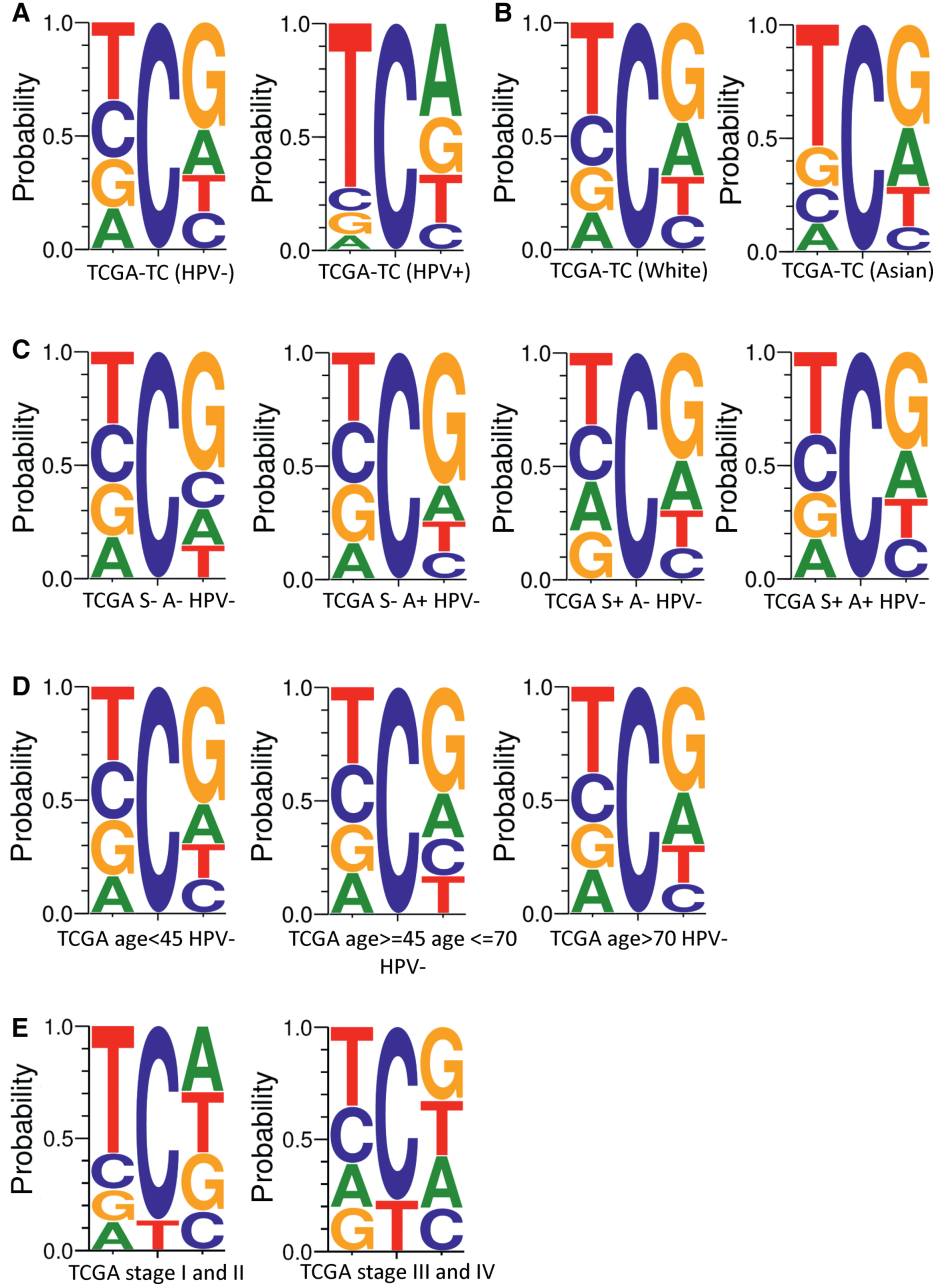
The context of the C>T mutations in the coding regions of the tongue carcinomas from TCGA project (TCGA‐TC) samples. A‐E, The context of the C>T mutations in the coding regions of the TCGA‐TC samples with different HPV statuses (A), races (B), smoking statuses (C), drinking statuses (C), ages (D), and stages (E)

To confirm whether the GCG pattern was unique to BQ‐TC or it is associated with some other factor, we analyzed the mutational signature in the 82 TCGA‐TC cases classified by different clinical parameters including HPV status, race, smoking, drinking, age, and stage. The HPV‐negative (HPV−) TCGA‐TC cases showed a TCG>TTG pattern, while the HPV‐positive (HPV+) TCGA‐TC cases had a strong preference for a T in the base preceding the mutated C residue of the C>T mutations (Figure 2A). Neither of them harbored the GCG>GTG pattern of BQ‐TC samples, which were all HPV‐negative detected by reverse dot blot and WGS analysis (Figure S4A‐B and Table S10). Furthermore, The GCG>GTG pattern was absent in any of the subgroups classified by race, smoking, drinking, age, or stage in TCGA‐TC (HPV+) cases (Figure 2B‐D).

### 3.3 The mutational signature in individual BQ‐TC cases

**Figure 3:**
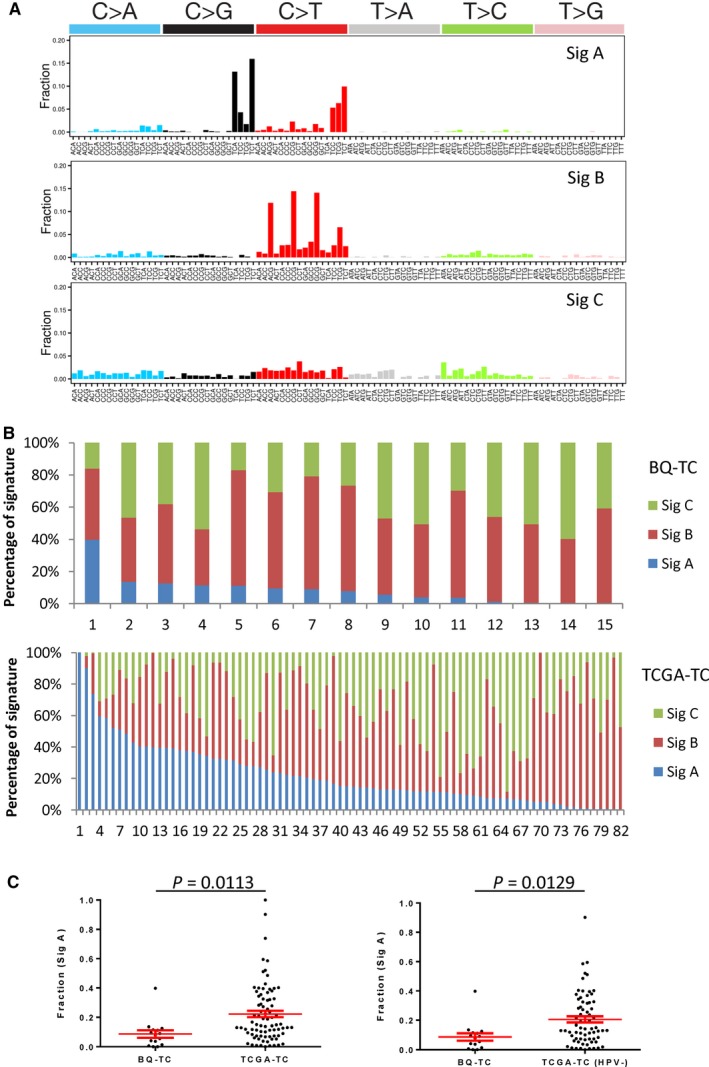
Mutational signature analysis of 102 tongue carcinoma (15 betel quid chewing‐associated tongue carcinoma [BQ‐TC], five nonbetel quid chewing‐associated tongue carcinoma [nBQ‐TC], and 82 tongue carcinomas from TCGA project [TCGA‐TC]) genomes using the Wellcome Trust Sanger Institute mutational signatures framework. A, The patterns of the three signatures (signature A, signature B, and signature C) observed in the 15 BQ‐TC, five nBQ‐TC, and 82 TCGA‐TC genomes, using the Wellcome Trust Sanger Institute mutational signatures framework. B, The proportion of signatures observed in the 15 BQ‐TC and 82 TCGA‐TC samples. C, Comparison of the proportion of signature A in the BQ‐TC and TCGA‐TC (HPV‐negative) samples (unpaired *t* test, two‐sided)

The mutational signature of 15 BQ‐TC, five nBQ‐TC, and 82 TCGA‐TC was analyzed using the Wellcome Trust Sanger Institute mutational signatures framework (Figure S5A). Signature A (Sig A), signature B (Sig B), and signature C (Sig C) were identified from 102 tongue carcinoma samples (Figure 3A). We conducted unsupervised hierarchical clustering of Sig A, Sig B, and Sig C with 30 mutational signatures (Sig 1‐30) previously identified in a pan‐cancer study^24^ using cosine similarity (Figure S5B). Sig A (the TCN pattern) is similar to Sig 13 (cosine similarity of 81.2%), which could be attributed to activity of the AID/APOBEC family of cytidine deaminases. Sig B (the GCG>GTG pattern) is similar to Sig 1 (Figure S5B) with a cosine similarity of 93.4%. Sig C is similar to Sig 5 with a cosine similarity of 89.5% (Figure S5B). The average proportion of Sig B in the BQ‐TC tumors is 53% (35%‐72%), and it is the dominant signature in BQ‐TC (Figure 3B). The proportion of Sig A in BQ‐TC (9%) is significantly lower than that in TCGA‐TC (22%) (*P* = 0.0113, unpaired *t* test, two‐sided) (Figure 3C). All the BQ‐TC samples are HPV‐negative, while a significant proportion of TCGA samples are HPV‐positive. To exclude the influence of HPV infection on the signature proportion, we determined the proportion of Sig A in HPV‐negative TCGA‐TC samples (21%), which was similar to HPV‐positive TCGA samples and significantly higher than that in the BQ‐TC samples (*P* = 0.0129, unpaired *t* test, two‐sided) (Figure 3C).

Since the BQ‐TCs are young cases compared with TCGA‐TC or nBQ‐TC (Table S2). Age can influence the mutation signature we analyzed, but this influence is limited (Figure 2D). As shown in Figure 2D, the proportion of T (but not G) preceding the mutated C is still dominating in the TCGA‐TC (age <45, HPV‐). Furthermore, we compared the Sig A proportion between BQ‐TC and the TCGA‐TC cases of age <60 (mean age = 46.3 years, similar with BQ‐TC). The proportion of Sig A of BQ‐TC is still lower than TCGA‐TC (*P* = 0.0037, unpaired *t* test, two‐sided, Figure S6). So the lower proportion of Sig A in BQ‐TC is not because of age, but the high proportion of Sig B in BQ‐TC.

### 3.4 The mutational spectrum of the BQ‐TC cases

**Figure 4:**
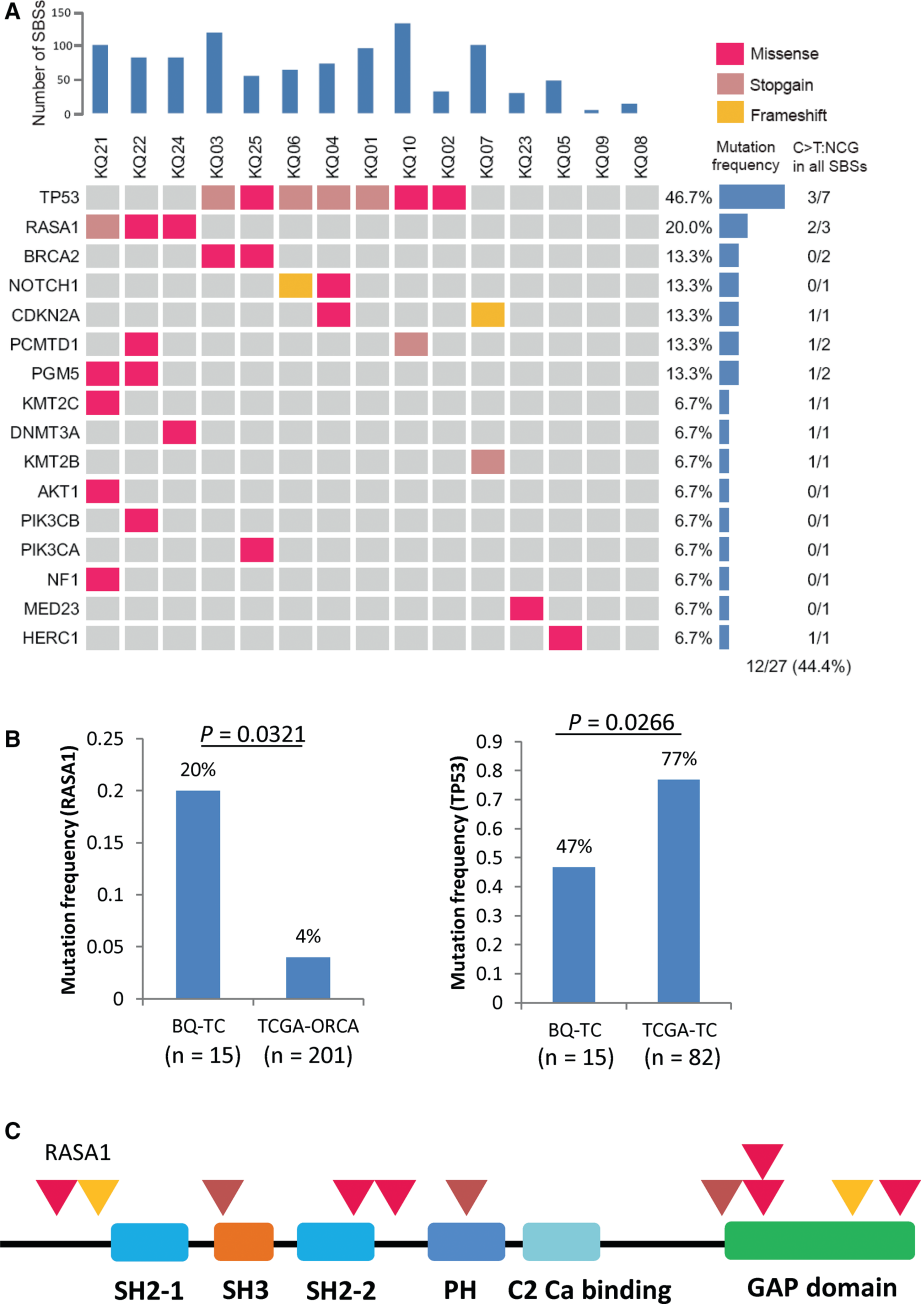
The mutational spectrum of the betel quid chewing‐associated tongue carcinomas (BQ‐TC) samples. A, The mutational spectrum of the BQ‐TC samples, showing number of somatic mutations in each tumor (top), the mutation frequency of each gene (right), and the proportion of C>T:NCG (C>T:ACG, C>T:CCG, C>T:GCG, C>T:TCG) in all SBSs of each genes (right). B, The mutation frequency of *RASA1* and *TP53* between BQ‐TC and either TCGA‐ORCA or TCGA‐TC from the general population. TCGA‐ORCA: oral carcinoma from the TCGA project. The “n” in the figure indicates the number of samples. Fisher's exact test, two‐sided. C, Diagrams of the mutations in the *RASA1* gene of tongue carcinoma. Red trilateral, missense mutation; gray red trilateral, stop‐gain mutation; yellow trilateral, frameshift mutation. All the *RASA1* gene nonsynonymous mutations in tongue carcinoma are summarized in Table S13

The BQ‐TC samples displayed a characteristic pattern of mutated genes. Among them, *TP53* and *RASA1 *have the top two mutation frequencies in the BQ‐TC cases (Figure 4A). *TP53* had the highest mutation frequency in the BQ‐TC cases and has also been reported to have a high mutation frequency in previous studies on head and neck carcinomas from the general population (Figure 4A and Table S11).^34^ However, the mutation frequency of *TP53* in BQ‐TC (47%) is lower than that in TCGA‐TC (77%) (*P* = 0.0266, Fisher's exact test, two‐sided) (Figure 4B and Table S12). Three out of the 15 BQ‐TC cases harbored RASA1 mutations, while there were only four *RASA1* mutations in the 82 TCGA cases and nine in the 201 TCGA‐ORCA cases (oral carcinoma from TCGA project). The mutation frequency of *RASA1* was higher in BQ‐TC (20%) compared with that in TCGA‐ORCA (4%) (*P* = 0.0321, Fisher's exact test, two‐sided, Figure 4B and Table S12). A stop‐gain mutation in RASA1 and a LOH in the other allele were identified in sample KQ21, indicating the tumor suppressor nature of RASA1 in TC (Figure 4A‐C and S7A). Furthermore, we analyzed the expression of *RASA1 *and found that it was downregulated in *RASA1*‐mutant TCGA‐ORCA samples compared with *RASA1* wild‐type TCGA‐ORCA samples (*P* = 0.0037, unpaired *t* test, two‐sided) (Figure S8A). *RASA1* expression levels in *RASA1* wild‐type tumors were similar to those in the matched normal tissues (*P* = 0.9957, paired *t* test, two‐sided, Figure S8B).

**Figure 5:**
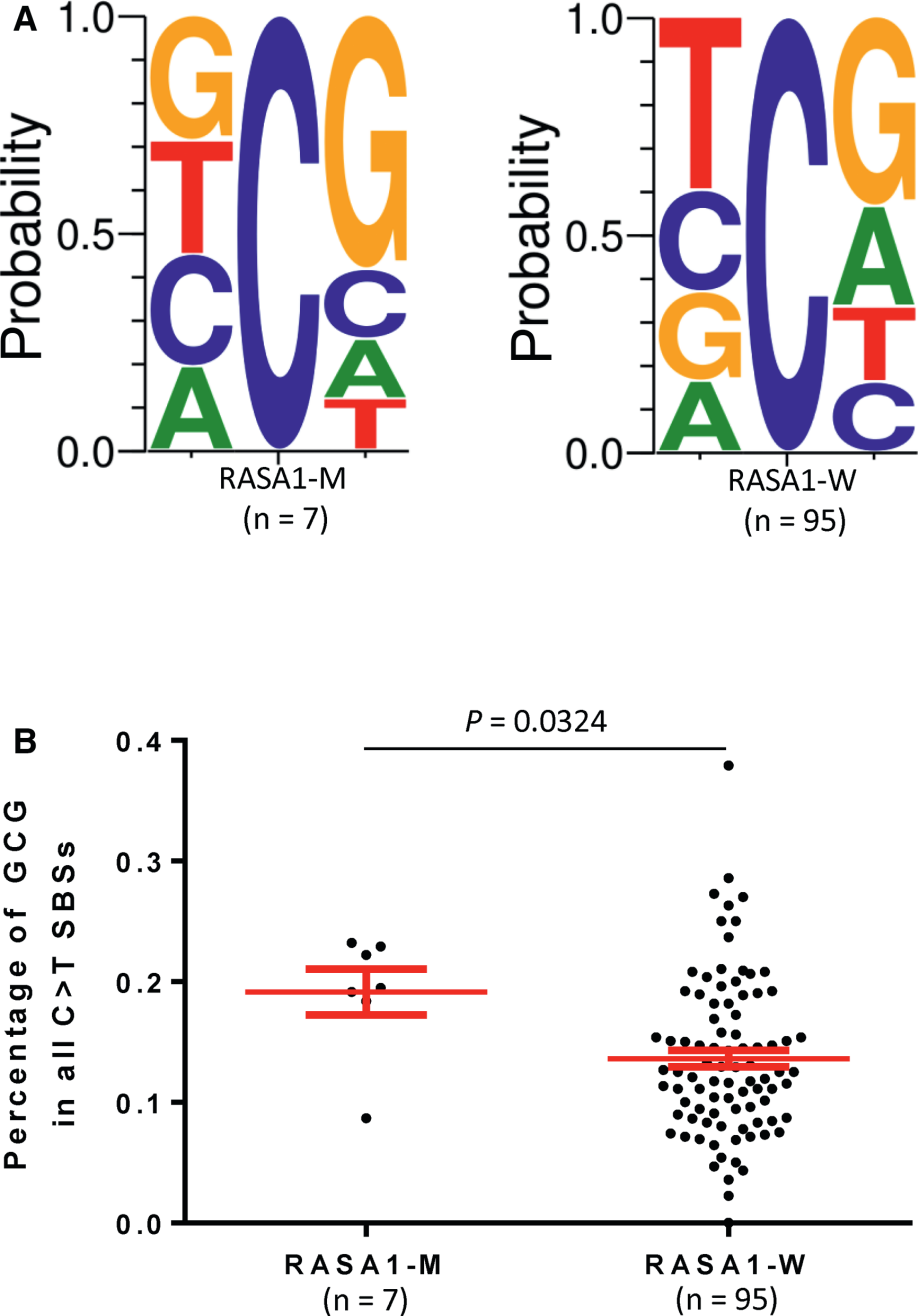
The mutational signature between *RASA1* mutation samples and *RASA1* wild‐type samples of tongue carcinomas. A‐B, The mutational signature between RASA1‐M (*RASA1* mutation samples from 102 tongue carcinomas) and RASA1‐W samples (*RASA1* wild‐type samples from 102 tongue carcinomas). The “n” in the figure indicates the number of samples. Unpaired *t* test, two‐sided

We further tested whether the *RASA1* mutations were associated with any genetic features. Altogether we identified seven samples harboring *RASA1* nonsynonymous mutations in the 102 tongue carcinomas that we analyzed (15 BQ‐TC, 5 nBQ‐TC, and 82 TCGA‐TC). The GCG (to GTG) pattern is the dominant pattern in these seven *RASA1* mutant cases, while in the rest of the 95 tongue carcinoma samples, TCG (to TTG) is the dominant pattern (Figure 5A). The proportion of the GCG (to GTG) pattern in *RASA1* mutant samples is significantly higher than that in the *RASA1* wild‐type samples (*P* = 0.0324, unpaired *t* test, two‐sided) (Figure 5B). Furthermore, we found that samples harboring *RASA1* or *TP53* mutations had higher count of copy number gains in the five BQ‐TC and five nBQ‐TC samples (*P* = 0.0152, Fisher's exact test, two‐sided) (Figure S7A‐B).

### 3.5 Frequent mutations in CpG islands

**Figure 6:**
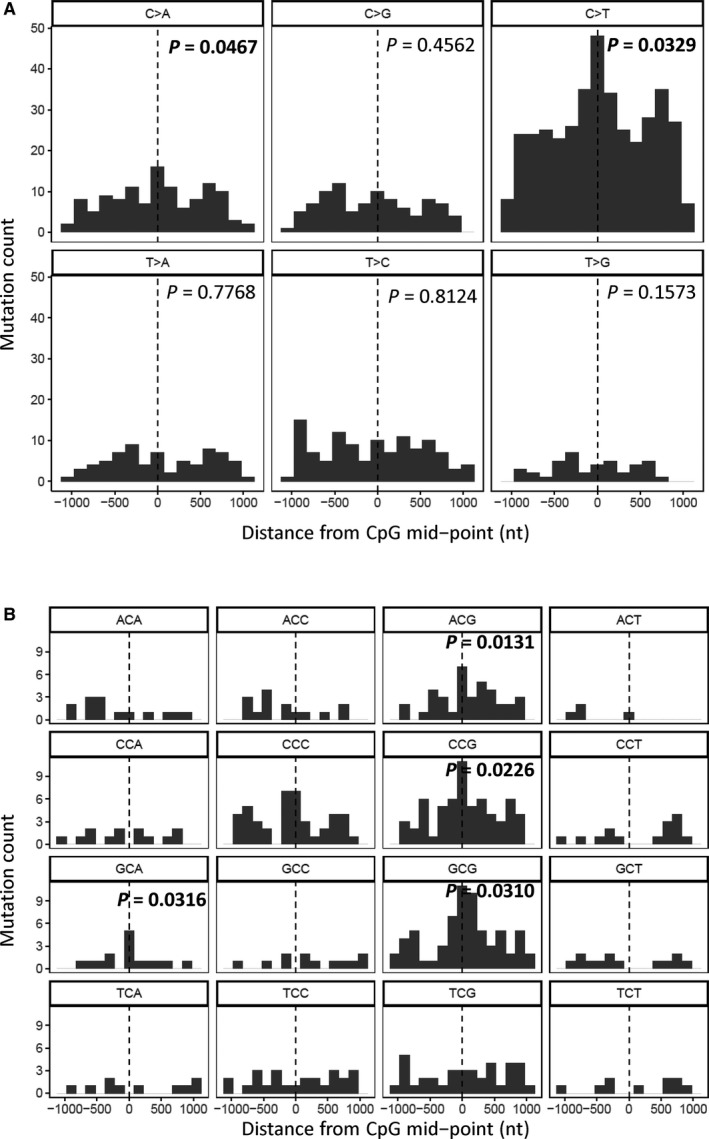
Mutation count in CpG islands of betel quid chewing‐associated tongue carcinomas (BQ‐TC) samples. A, Mutation count of SBSs in the six mutation classes in CpG islands and their flanking regions in five BQ‐TC samples with whole‐genome sequencing. B, Mutation count of C>T SBSs in the 16 mutation classes (according to the context of the C>T mutations) in CpG islands and their flanking regions in five BQ‐TC samples with whole‐genome sequencing (only show *P* < 0.05). The relative positions of the CpG islands are labeled on the *x*‐axis. *P*‐value was calculated using Fisher's exact test, two‐sided. *P* values <0.05 are in bold.

We analyzed the WGS data to identify mutations in noncoding regions. We found highly enriched mutations in the CpG islands throughout the whole genome of BQ‐TC, but not in that of nBQ‐TC samples (Figure 6A and S9A‐B). The mutation rate in the CpG islands is 2.05 times higher than that in the matched flanking regions (*P* = 0.0467, Fisher's exact test, two‐sided). The C>T mutations are the major contributor of this enrichment and provided 48% of the mutations in CpG islands. The NCG>NTG signature is the most significant subtype of this mutation class (Figure 6B). No significant enrichment has been identified in other noncoding region such as transcription factor binding sites.

## 4 DISCUSSION

Tongue cancer is a common malignancy of the oral cavity, and the risk factors for tongue cancer include tobacco (smoking and chewing), alcohol, and betel product chewing.^7^ Hunan Province is one leading consumer of betel‐related products. The incidence of tongue carcinoma in Hunan is much higher than that in other regions of China, and most tongue carcinoma patients in Hunan have the habit of BQ chewing. Different from Hainan or Taiwan, areca tree does not grow in Hunan and nearly no fresh BN is available in Hunan. BQ is the major form of betel consumption in Hunan, and the BQ in Hunan does not have tobacco mixed. In this case, the tongue carcinoma samples in Hunan provide a chance to study the effect of BQ on OC without the affection of tobacco chewing. We analyzed the genome of 15 BQ‐TC and compared their genetic features with five nBQ‐TC in China and 82 nBQ‐TC from TCGA database. We identified a characteristic mutational signature and genetic landscape in BQ‐TC, which was distinct from the tongue carcinomas in general population.

Exposure to exogenous mutagens leads to an increased mutation load and a special mutational signature on the genome, which is associated with the molecular mechanism of the mutagenic process.^23^, ^24^, ^35^ BQ chewing is strongly associated with oral pre‐cancer and OC.^6^ Our genome‐wide sequencing data do not support the mutagenic mechanism of BQ chewing as there is not an increased mutation load in the BQ‐TC cases compared with tongue carcinomas from the general population. However, the genetic feature of BQ‐TC does distinguish it as a unique subtype of tongue carcinoma. The GCG (to GTG) pattern of the C>T mutations is the dominant mutation pattern in BQ‐TC, but not in TCGA‐TC. This association still exists when we check the samples by age, smoking, HPV infection, etc^24^ We did not observe any mutational signature associated with smoking or tobacco chewing.

Carcinogens influence mutational signatures through direct (mutagens, such as UV and smoke, with a high mutation load) and indirect mechanisms (DNA editing by APOBEC, altering DNA methylation, and other mechanisms).^36^ Based on our comparison, BQ chewing‐induced tongue carcinoma exhibited a similar mutational level compared with Helicobacter pylori‐associated gastric cancer. This finding suggests that BQ chewing may not act via a direct mechanism (as a mutagen, such as UV and smoke) but rather via an indirect mechanism. Furthermore, we observed that BQ chewing was related to DNA methylation (but not DNA editing by APOBEC, Figure S8), thus supporting the indirect role in the mutational signature change.

We observed differences in the mutational landscape between BQ‐TC and tongue carcinomas from the general population. RAS signaling pathway changes, especially *RASA1* mutations, are significantly frequent in BQ‐TC cases. RASA1 acts as a suppressor of RAS function by enhancing the GTPase activity of RAS proteins, which can transform RAS to the inactive GDP‐bound form.^37^ A *RASA1* stop‐gain mutation and a LOH were observed in the same BQ‐TC sample, indicating that *RASA1* functions as a tumor suppressor gene, which might play an important role in RAS signaling pathway activation and contribute to tumorigenesis of BQ‐TC.

More and more tumor types have been studied in the whole‐genome scale. However, less driver mutations have been identified in the noncoding regions than those in the coding regions. In this study, we did not identify any recurrent hot spot mutation like that in the promoter region of *TERT*.^38^ However, we found frequent mutations in the CpG islands. No enriched mutations have been identified in other noncoding regions like transcription factor binding regions.^39^ The mutations in CpG islands could change the methylation status and then gene expression levels to contribute to the tumorigenesis. Interestingly, the GCG>GTG signature is also the major contributor of the CpG island mutations.

Collectively, we characterized the genomic features of BQ‐TC. The characteristic mutational signature of BQ‐TC is the major contributor of the frequent mutations in RASA1 and CpG islands, which could drive the tumorigenesis by activating RAS signaling pathway and changing methylation status. The mutational signature and landscape define BQ‐TC as a subtype of tongue carcinoma. Our study provides new insights into the carcinogenic mechanism of BQ chewing.

## CONFLICT OF INTEREST

None declared.
