# Supplementary material for: Mutational signatures and the genomic landscape of betel quid chewing‐associated tongue carcinoma
Source: Cancer Med. 2019 Jan 22;8(2):701–11. doi: 10.1002/cam4.1888 (PMC6382727; doi:10.1002/cam4.1888)
Supplement: Supplementary file 1 [file CAM4-8-701-s001.pdf]

Fig. S1

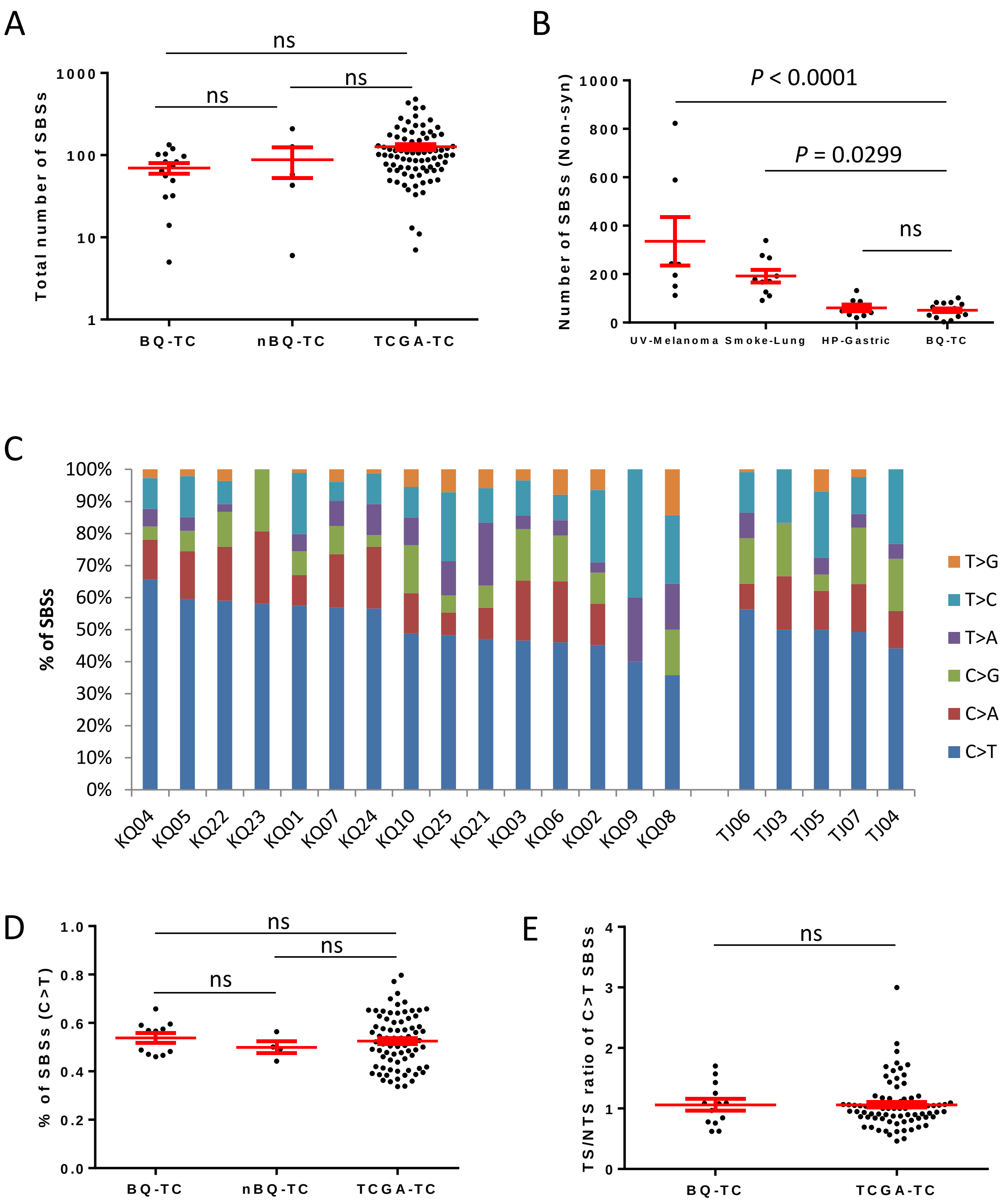

A
ns
ns
ns
Total number of SBSs
1000
100
10
1
BQ-TC
nBQ-TC
TCGA-TC
B
P < 0.0001
P = 0.0299
ns
Number of SBSs (Non-syn)
1000
800
600
400
200
0
UV-Melanoma
Smoke-Lung
HP-Gastric
BQ-TC
C
% of SBSs
100%
90%
80%
70%
60%
50%
40%
30%
20%
10%
0%
KQ04
KQ05
KQ22
KQ23
KQ01
KQ07
KQ24
KQ10
KQ25
KQ21
KQ03
KQ06
KQ02
KQ09
KQ08
TJ06
TJ03
TJ05
TJ07
TJ04
T>G
T>C
T>A
C>G
C>A
C>T
D
ns
ns
ns
% of SBSs (C>T)
1.0
0.8
0.6
0.4
0.2
0.0
BQ-TC
nBQ-TC
TCGA-TC
E
ns
TS/NTS ratio of C>T SBSs
4
3
2
1
0
BQ-TC
TCGA-TC

Fig. S1

Fig. S1

The mutational signature of individual BQ-TC and nBQ-TC samples.

A, Mutation counts in the whole exome of 15 BQ-TC, 5 nBQ-TC and 82 TCGA-TC samples. One-way ANOVA. B, The nonsynonymous mutation load of BQ-TC cases compared with three tumor types associated with exposure to Group 1 human carcinogens. One-way ANOVA. C, Distribution of the six mutation classes in the 15 BQ-TC and 5 nBQ-TC samples. D, Scatter plot of the percentage of C>T mutations in all SBS mutations of BQ-TC, nBQ-TC and TCGA-TC samples. One-way ANOVA. E, Scatter plot of the strand bias of C>T mutations in the coding regions of the BQ-TC, nBQ-TC and TCGA-TC samples. Unpaired t test, two sided. TS: transcribed strand; NTS: nontranscribed strand.

Data were expressed as mean $\pm$ SEM in scatter plot of this paper. ns: *P*-value is not smaller than 0.05, no statistical significance.

Fig. S2

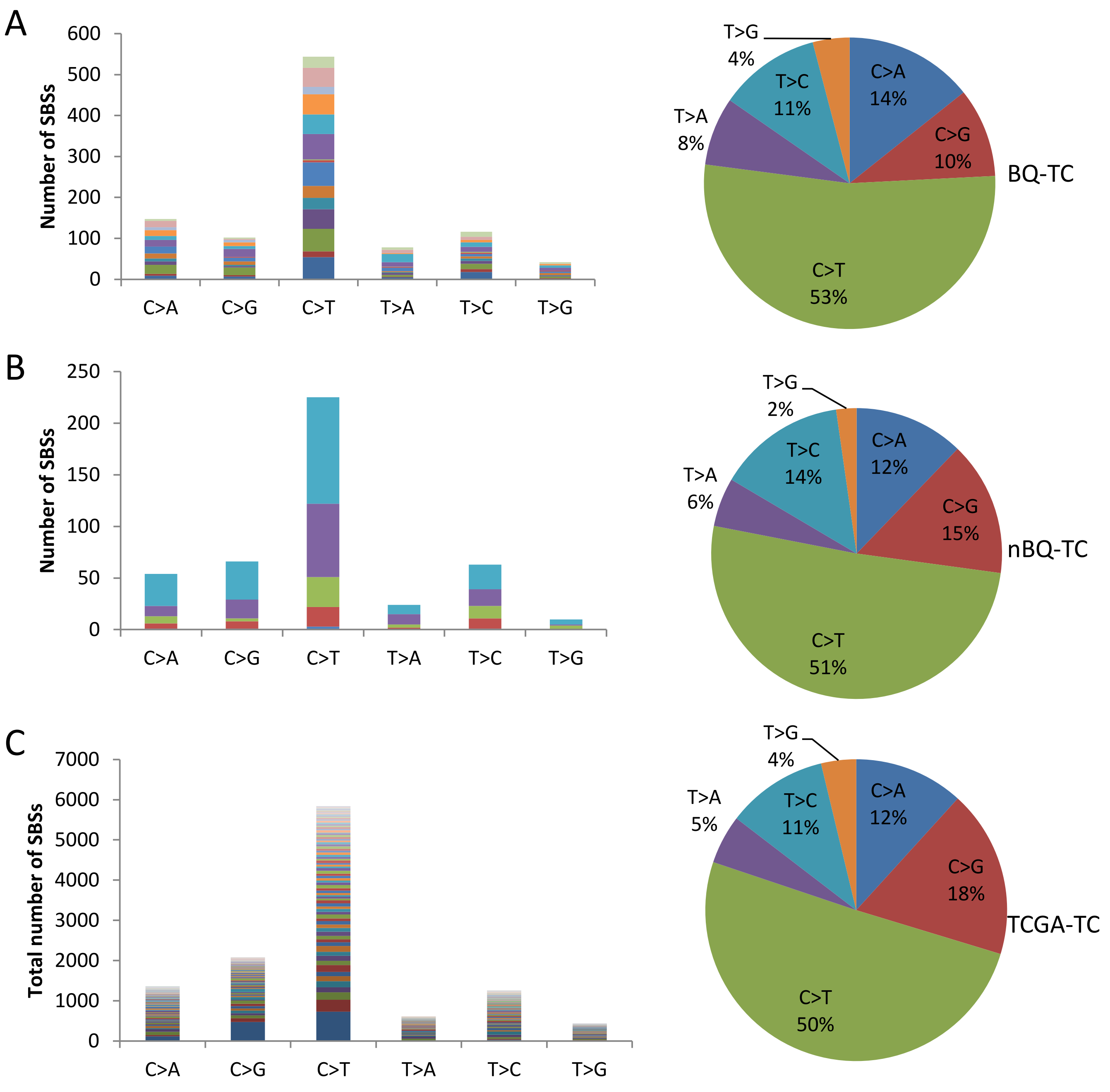
A
Number of SBSs
600
500
400
300
200
100
0
C>A
C>G
C>T
T>A
T>C
T>G
T>G
4%
T>C
11%
T>A
8%
C>A
14%
C>G
10%
C>T
53%
BQ-TC
B
Number of SBSs
250
200
150
100
50
0
C>A
C>G
C>T
T>A
T>C
T>G
T>G
2%
T>C
14%
T>A
6%
C>A
12%
C>G
15%
C>T
51%
nBQ-TC
C
Total number of SBSs
7000
6000
5000
4000
3000
2000
1000
0
C>A
C>G
C>T
T>A
T>C
T>G
T>G
4%
T>C
11%
T>A
5%
C>A
12%
C>G
18%
C>T
50%
TCGA-TC

Fig. S2

Fig. S2

Mutational signature of BQ-TC, nBQ-TC and TCGA-TC samples.

A, Percentage of the six possible mutation classes in the 15 BQ-TC samples. B, Percentage of the six possible mutation classes in the 5 nBQ-TC samples. C, Percentage of the six possible mutation classes in the 82 TCGA-TC samples.

Fig. S3

A

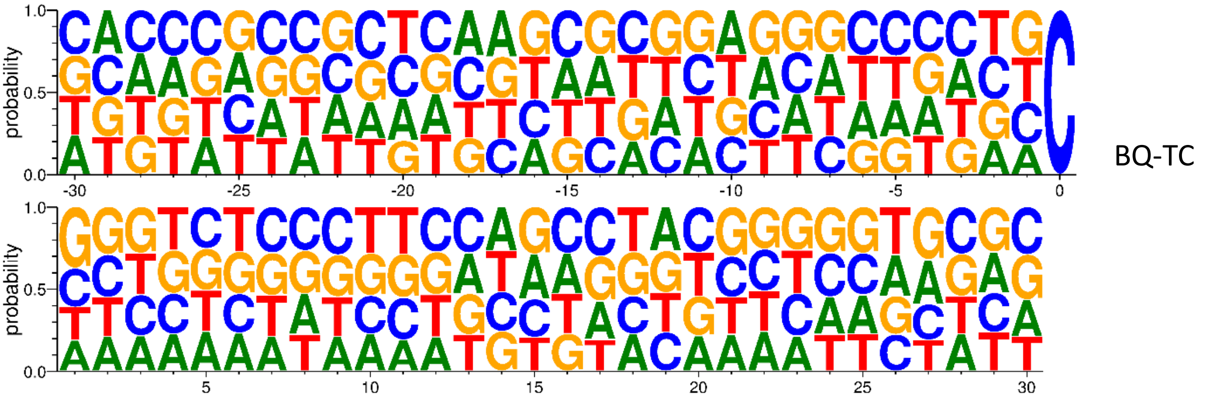

B

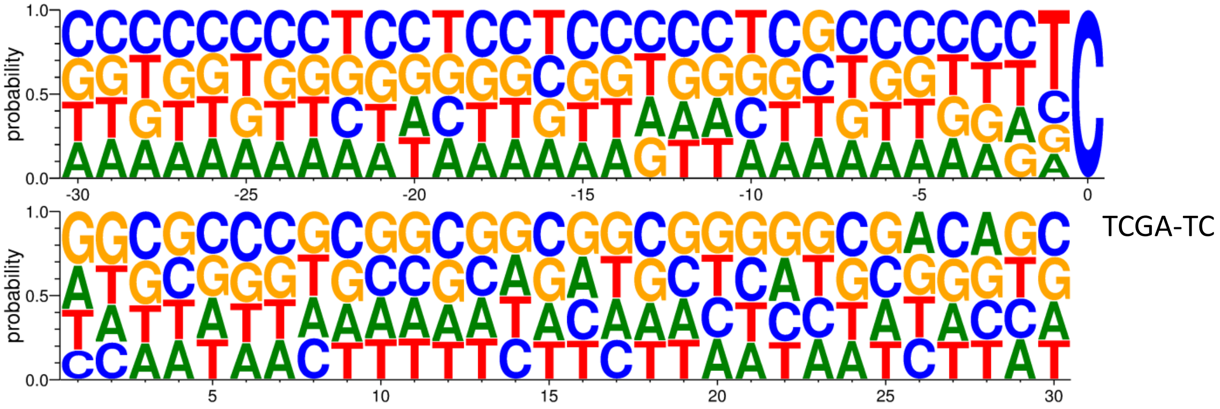

Fig. S3

Fig. S3
The context of C>T mutations BQ-TC and TCGA-TC genome.
A, The 5’context (30 bases) and 3’context (30 bases) of C>T mutations in 15 BQ-TC samples. B, The 5’context (30 bases) and 3’context (30 bases) of C>T mutations in 82 TCGA-TC samples.

Fig. S4

A

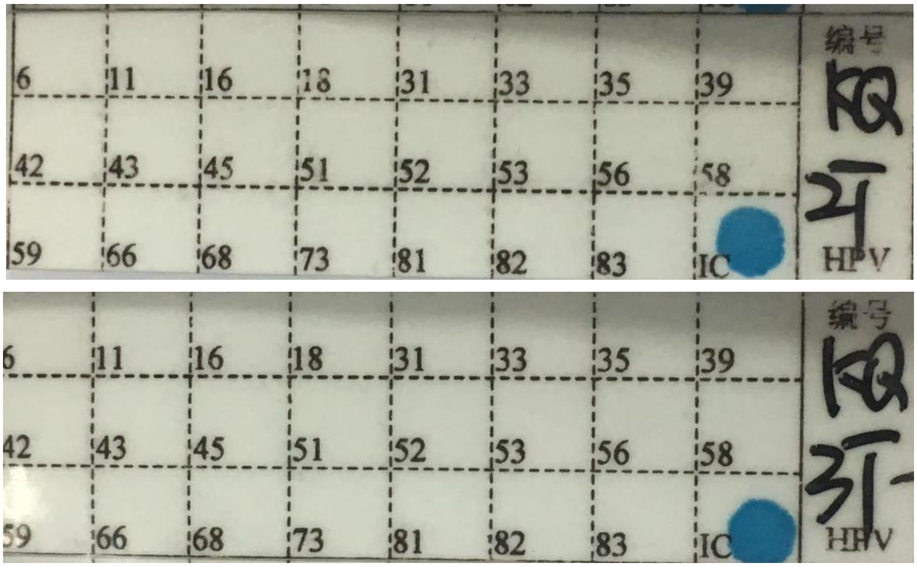

KQ02

KQ03

Figure S5.
Relative RNA expression of *RASA1* and APOBEC family genes in [illegible]inoma from TCGA project.
A, Relative RNA expression of *RASA1* between RASA1-M (*RASA1* [illegible] samples from TCGA-ORCA) and RASA1-W samples (*RASA1* wild-type samples from TCGA-ORCA). TCGA-ORCA: oral carcinoma from TCGA project. Unpaired t test, two sided. B, Relative RNA expression of *RASA1* between tumor and matched normal samples from TCGA-ORCA. Paired t test, two sided. C-D, Relative RNA expression of *APOBEC3A* (C) and *APOBEC3B* (D) between RASA1-M and RASA1-W samples. Unpaired t test, two sided. E, *P*-value of relative RNA expression of APOBEC family genes between RASA1-M and RASA1-W samples. Unpaired t test, two sided.

B

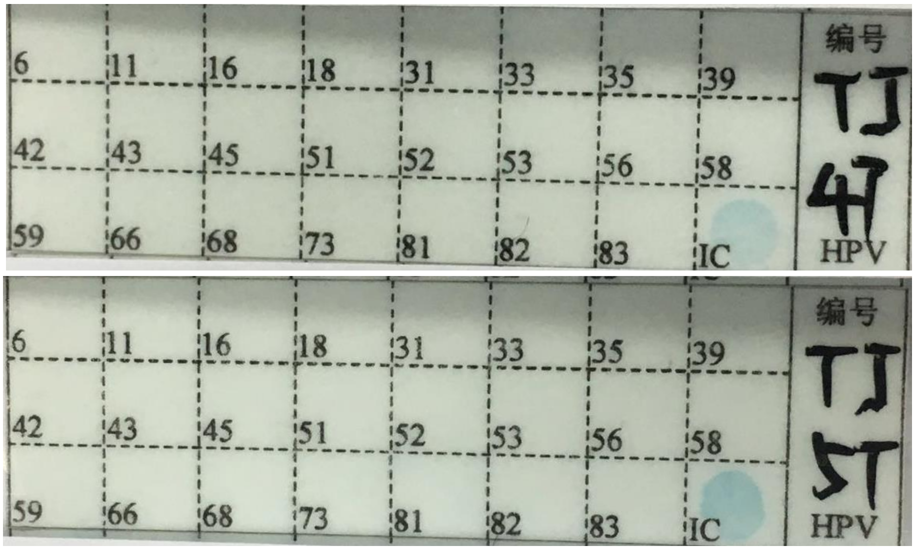

TJ04

TJ05

Fig. S4

Fig. S4

HPV detection in BQ-TC and nBQ-TC tumor samples through reverse dot blot.

A-B, HPV detection in BQ-TC (KQ02, KQ03) and nBQ-TC (TJ04, TJ05) tumor samples through reverse dot blot. The number means one type of HPV. HPV16, 18, 31, 33, 35, 39, 45, 51, 52, 53, 56, 58, 59, 66, 68, 73, 82 and HPV6, 11, 42, 43 ,81, 83 were detected (totally 23 type of HPV). IC, positive control, blue dot means positive.

Fig. S5

A

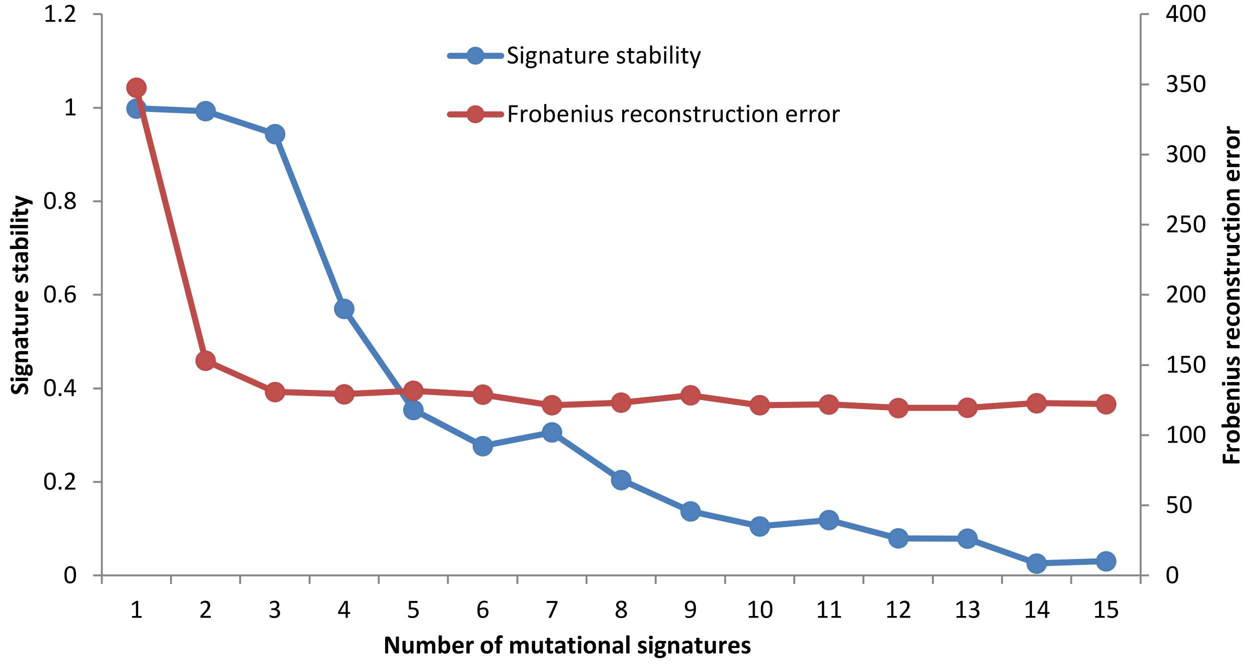

Signature stability
Frobenius reconstruction error
Signature stability
Frobenius reconstruction error
Number of mutational signatures
1.2
1
0.8
0.6
0.4
0.2
0
400
350
300
250
200
150
100
50
0
1 2 3 4 5 6 7 8 9 10 11 12 13 14 15

B

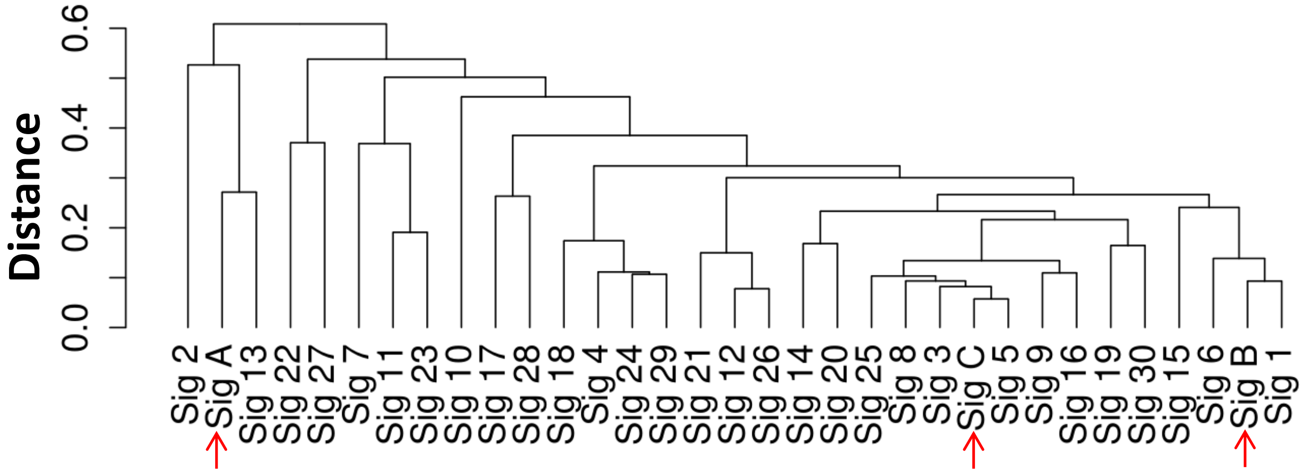

Distance
0.0
0.2
0.4
0.6
Sig 2
Sig A
Sig 13
Sig 22
Sig 27
Sig 7
Sig 11
Sig 23
Sig 10
Sig 17
Sig 28
Sig 18
Sig 4
Sig 24
Sig 29
Sig 21
Sig 12
Sig 26
Sig 14
Sig 20
Sig 25
Sig 8
Sig 3
Sig C
Sig 5
Sig 9
Sig 16
Sig 19
Sig 30
Sig 15
Sig 6
Sig B
Sig 1

Fig. S5

Fig. S5
Mutational signature analysis of 102 tongue carcinoma genomes using using the Wellcome Trust Sanger Institute mutational signatures framework.
A, Identifying the number of processes operative in the 15 BQ-TC, 5 nBQ-TC and 82 TCGA-TC samples based on reproducibility of their signatures and low error for re-constructing obtained for K = 1 to 15 signatures. B, Unsupervised hierarchical clustering of 3 mutational signatures identified in our series (Sig A Sig B and Sig C) and 30 mutational signatures previously identified in a pan-cancer study (Sig 1-30).

Fig. S6

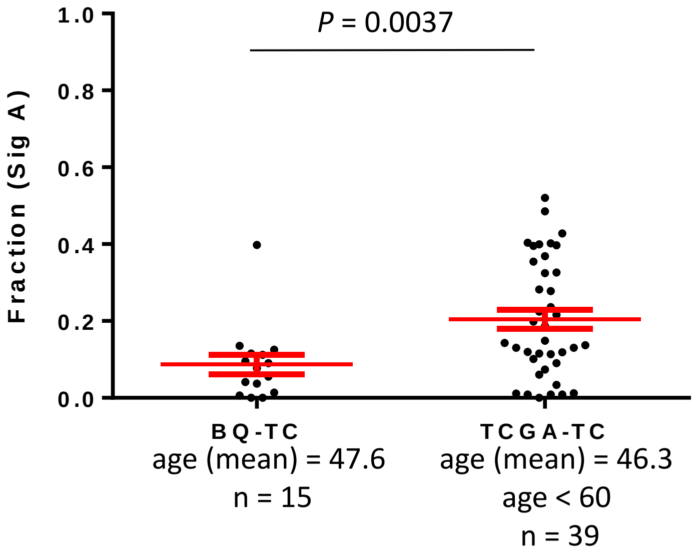
P = 0.0037
Fraction (Sig A)
1.0
0.8
0.6
0.4
0.2
0.0
BQ-TC
age (mean) = 47.6
n = 15
TCGA-TC
age (mean) = 46.3
age < 60
n = 39

Fig. S6

Fig. S6
We compared the Sig A proportion between BQ-TC and the TCGA-TC cases of age<60.

A, we compared the Sig A proportion between BQ-TC and the TCGA-TC cases of age<60 (mean age = 46.3 years, similar with BQ-TC).

Fig. S7

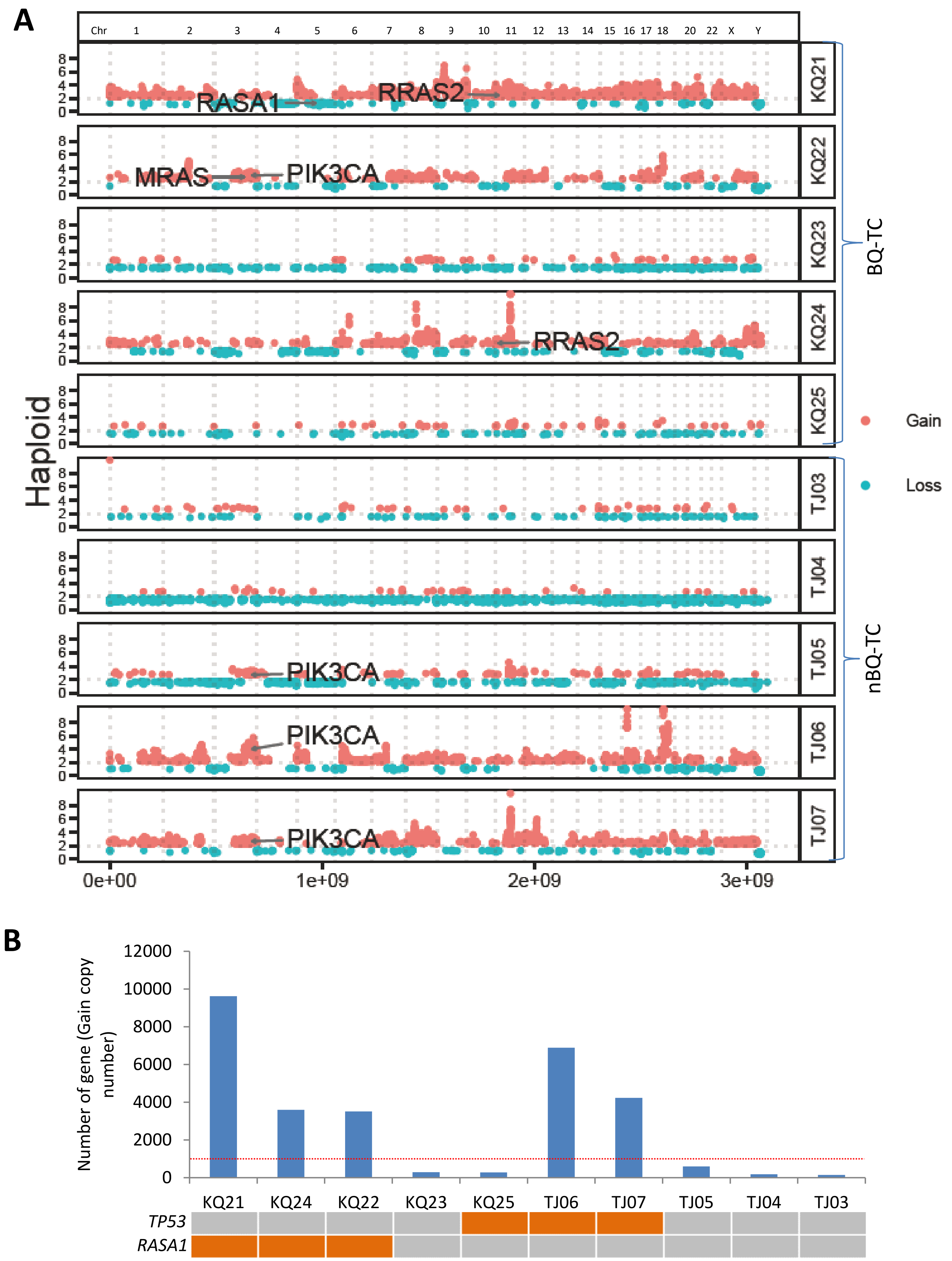

A
Chr 1 2 3 4 5 6 7 8 9 10 11 12 13 14 15 16 17 18 20 22 X Y
KQ21
KQ22
KQ23
KQ24
KQ25
TJ03
TJ04
TJ05
TJ06
TJ07
RASA1
RRAS2
MRAS
PIK3CA
Haploid
BQ-TC
nBQ-TC
Gain
Loss
0e+00
1e+09
2e+09
3e+09
B
Number of gene (Gain copy number)
12000
10000
8000
6000
4000
2000
0
KQ21 KQ24 KQ22 KQ23 KQ25 TJ06 TJ07 TJ05 TJ04 TJ03
TP53
RASA1

Fig. S7

C

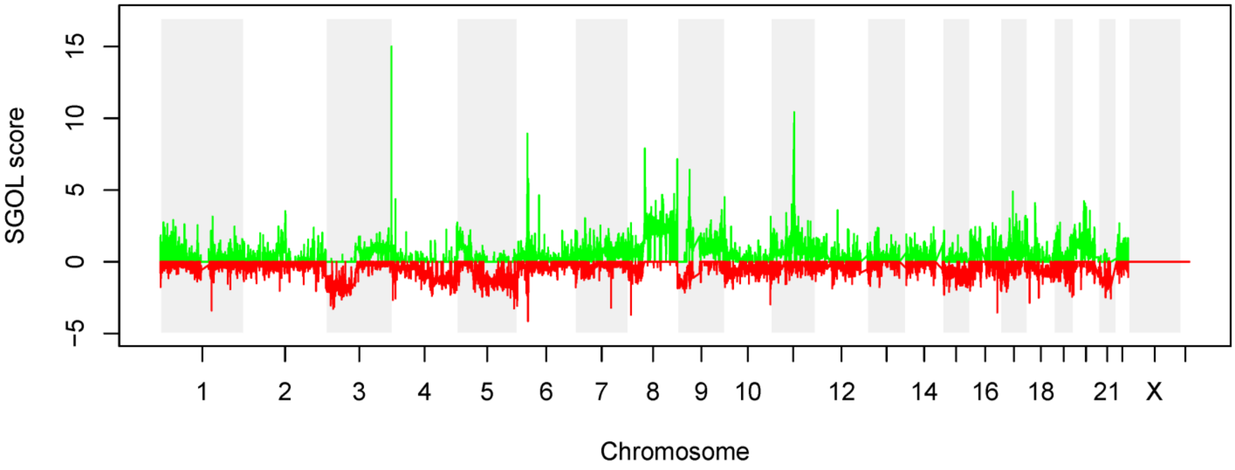

BQ-TC

D

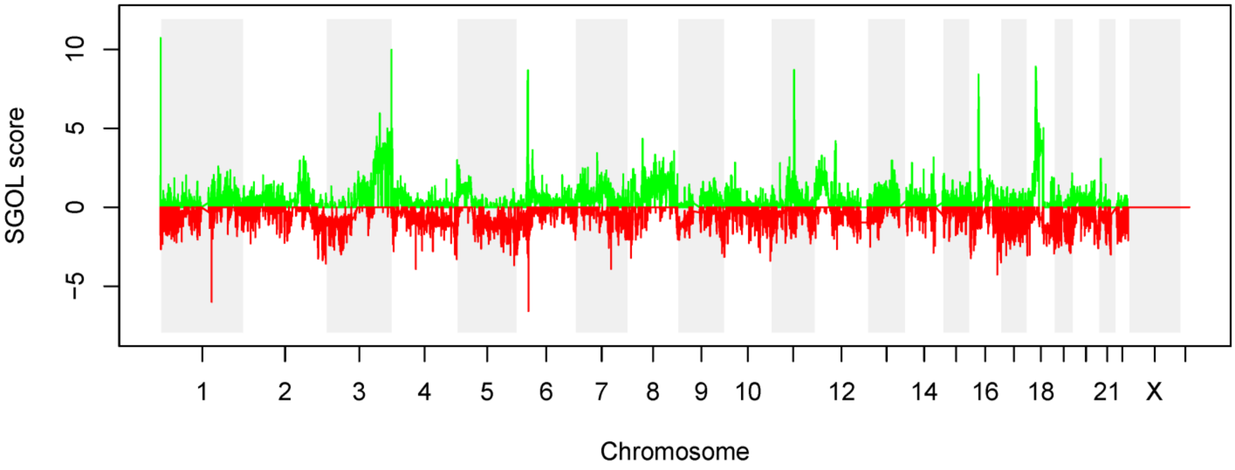

nBQ-TC

Fig. S7

Fig. S7
Landscape of the genomic somatic copy number alterations in the 5 BQ-TC and 5 nBQ-TC samples with whole-genome sequencing.
A, the somatic copy number mutational spectrum in the 5 BQ-TC and 5 nBQ-TC samples determined by whole-genome sequencing. *RASA1*, *PIK3CA*, *KRAS*, *HRAS* and *NOTCH1* are indicated with arrows. B, the relationship between the number of genes with a gain of copy and the *TP53* and *RASA1* mutations in the 5 BQ-TC and 5 nBQ-TC samples determined by whole-genome sequencing. The red dashed line means y = 1000; > 1000 means high copy-number gain; < 1000 means low copy-number gain. The gray blocks indicate wild-type *TP53* or *RASA1* and the yellow blocks indicate mutant *TP53* or *RASA1*. The *P*-values of *TP53*/*RASA1* mutation status (*TP53*/*RASA1* mutation or *TP53*/*RASA1* wild-type) and copy number status (high copy-number gain and low copy-number gain) were calculated using Fisher's exact test, two-sided, *P* = 0.0152. C-D, recurrence of chromosomal alterations in BQ-TC and nBQ-TC from WGS. SGOL scores for each segmented data (y axis) plotted aligned along the x axis in genome order. Green represents chromosomal gain, and red denotes chromosomal loss.

Fig. S8

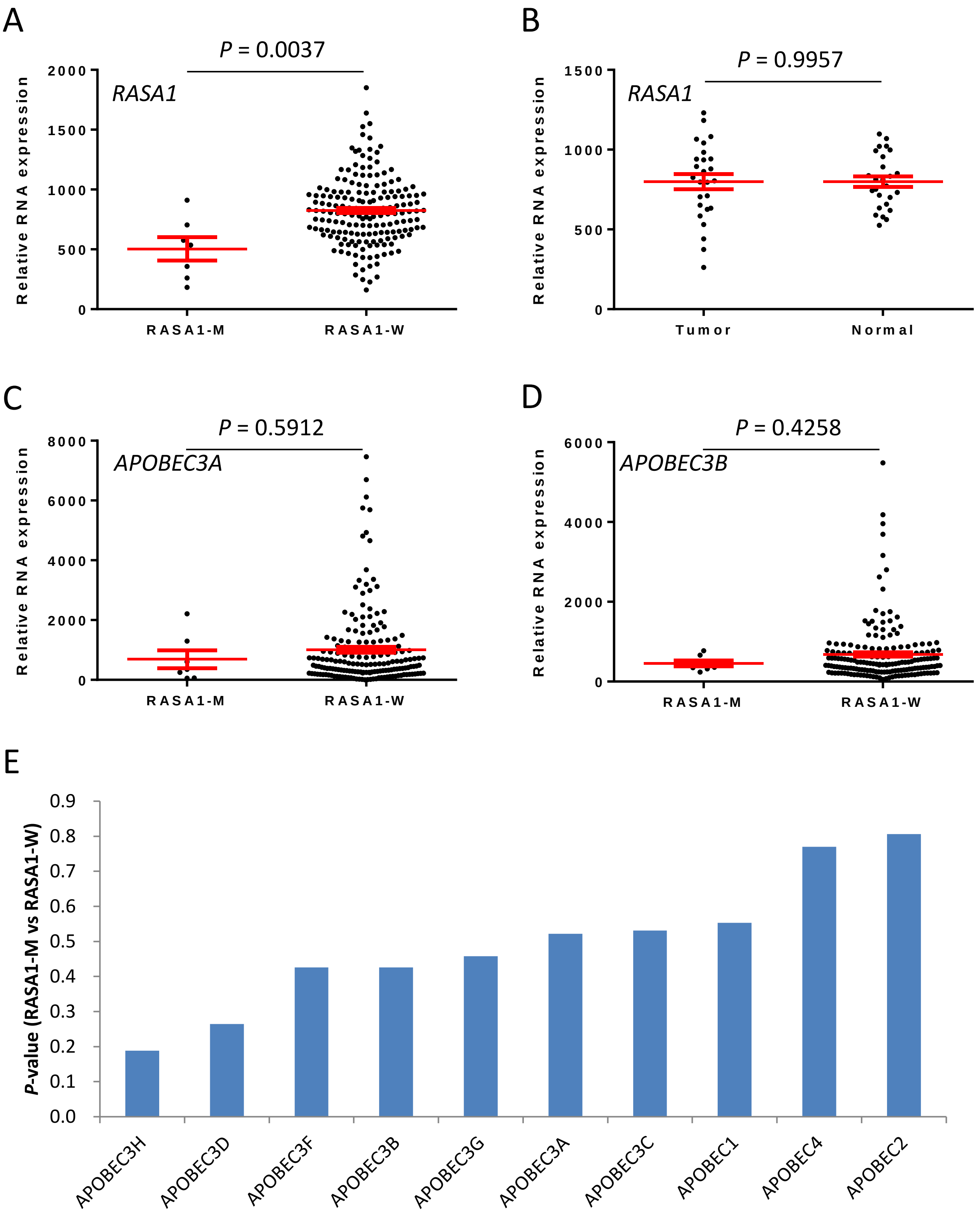

A
P = 0.0037
RASA1
Relative RNA expression
2000
1500
1000
500
0
RASA1-M
RASA1-W
B
P = 0.9957
RASA1
Relative RNA expression
1500
1000
500
0
Tumor
Normal
C
P = 0.5912
APOBEC3A
Relative RNA expression
8000
6000
4000
2000
0
RASA1-M
RASA1-W
D
P = 0.4258
APOBEC3B
Relative RNA expression
6000
4000
2000
0
RASA1-M
RASA1-W
E
P-value (RASA1-M vs RASA1-W)
0.9
0.8
0.7
0.6
0.5
0.4
0.3
0.2
0.1
0.0
APOBEC3H
APOBEC3D
APOBEC3F
APOBEC3B
APOBEC3G
APOBEC3A
APOBEC3C
APOBEC1
APOBEC4
APOBEC2

Fig. S8

Fig. S8
Relative RNA expression of *RASA1* and APOBEC family genes in oral carcinoma from TCGA project.
A, Relative RNA expression of *RASA1* between RASA1-M (*RASA1* mutation samples from TCGA-ORCA) and RASA1-W samples (*RASA1* wild-type samples from TCGA-ORCA). TCGA-ORCA: oral carcinoma from TCGA project. Unpaired t test, two sided. B, Relative RNA expression of *RASA1* between tumor and matched normal samples from TCGA-ORCA. Paired t test, two sided. C-D, Relative RNA expression of *APOBEC3A* (C) and *APOBEC3B* (D) between RASA1-M and RASA1-W samples. Unpaired t test, two sided. E, *P*-value of relative RNA expression of APOBEC family genes between RASA1-M and RASA1-W samples. Unpaired t test, two sided.

Fig. S9

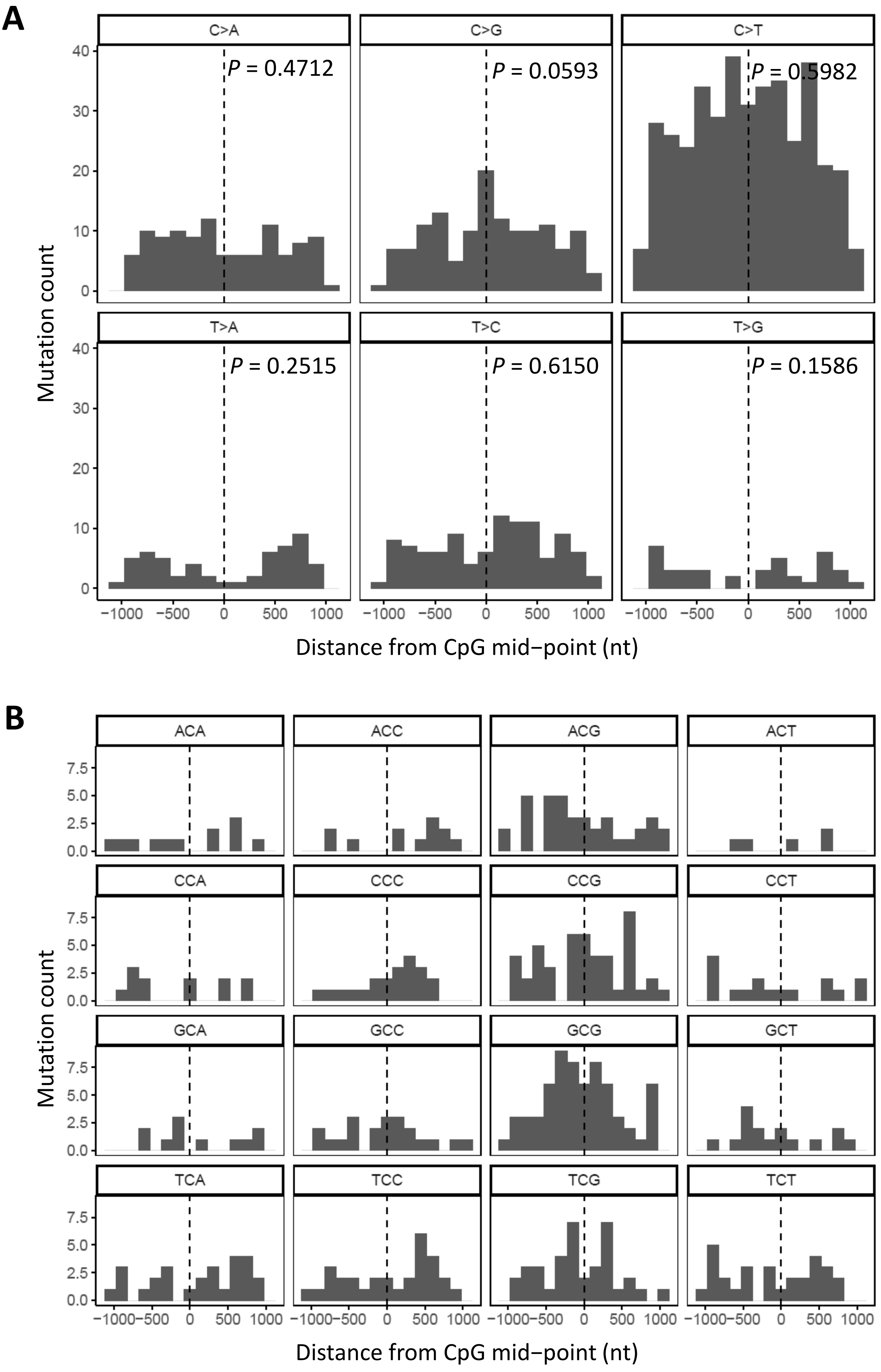
A
C>A
C>G
C>T
T>A
T>C
T>G
P = 0.4712
P = 0.0593
P = 0.5982
P = 0.2515
P = 0.6150
P = 0.1586
Mutation count
Distance from CpG mid−point (nt)
B
ACA
ACC
ACG
ACT
CCA
CCC
CCG
CCT
GCA
GCC
GCG
GCT
TCA
TCC
TCG
TCT
Mutation count
Distance from CpG mid−point (nt)

Fig. S9

Fig. S9

Mutation count in CpG islands of nBQ-TC samples.

A, mutation count of SBSs in the six mutation classes in CpG islands and their flanking regions in 5 nBQ-TC samples with whole genome sequencing. B, mutation count of C>T SBSs in the 16 mutation classes (according to the context of the C>T mutations) in CpG islands and their flanking regions in 5 nBQ-TC samples with whole genome sequencing (only show $P < 0.05$). The relative positions of the CpG islands are labeled on the X axis. *P*-value were calculated using Fisher's exact test, two-sided. $P < 0.05$ are in bold.
